# Nanopatterning with Photonic Nanojets: Review and Perspectives in Biomedical Research

**DOI:** 10.3390/mi12030256

**Published:** 2021-03-03

**Authors:** Salvatore Surdo, Martí Duocastella, Alberto Diaspro

**Affiliations:** 1 Nanoscopy, Istituto Italiano di Tecnologia, Via Enrico Melen 83, Building B, 16152 Genoa, Italy; 2 Department of Applied Physics, University of Barcelona, C/Martí i Franquès 1, 08028 Barcelona, Spain; 3 Department of Physics, University of Genoa, Via Dodecaneso 33, 16146 Genova, Italy

**Keywords:** nanotechnology, lithography, laser direct-write, near field, subwavelength

## Abstract

Nanostructured surfaces and devices offer astounding possibilities for biomedical research, including cellular and molecular biology, diagnostics, and therapeutics. However, the wide implementation of these systems is currently limited by the lack of cost-effective and easy-to-use nanopatterning tools. A promising solution is to use optical methods based on photonic nanojets, namely, needle-like beams featuring a nanometric width. In this review, we survey the physics, engineering strategies, and recent implementations of photonic nanojets for high-throughput generation of arbitrary nanopatterns, along with applications in optics, electronics, mechanics, and biosensing. An outlook of the potential impact of nanopatterning technologies based on photonic nanojets in several relevant biomedical areas is also provided.

## 1. Introduction

The ability to pattern a surface with tailored nanostructures, such as trenches, pillars, and spirals, has led to significant advances in the field of biomedical research [1,2,3]. Nanoscale features provide access to unique mechanical, optical, and electronic phenomena. As such, they allow the increase of the precision and control of artificially engineered tissues [4], biosensors [5], or drug-delivery systems [6]. However, the path toward a broader adoption of nanostructures in biology and biomedicine presents several barriers. Among them is the lack of simple, scalable, versatile, and cost-effective nanofabrication methods. For instance, conventional techniques, such as deep UV and electron beam lithography, can only operate with a scarce selection of materials, on planar geometries, and using rigid substrates. In contrast, maskless laser technologies are compatible with multiple materials [7] and substrates [8] and enable the generation of complex shapes [9,10] in both subtractive and additive modes [11,12]. Still, the minimum feature size and spatial resolution are typically dictated by the diffraction limit—above 100 nm for commercial laser wavelengths. 

**Figure 1:**
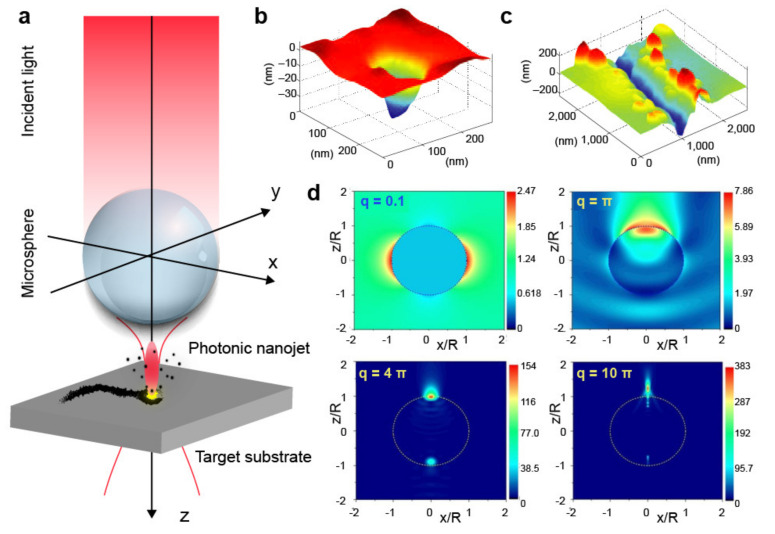
(**a**) Schematic description of photonic nanojet-mediated nanopatterning using a microsphere. (**b**,**c**) Atomic force microscope (AFM) topography maps of a nanohole (**b**) and a nanochannel (**c**) ablated on a polycarbonate substrate using a photonic nanojet and pulsed irradiation. Adapted with permission from [20]. Copyright 2008 Springer Nature. (**d**) Theoretical intensity distributions of the optical field generated by a dielectric microsphere ($n_{p}$ = 1.5 and $n_{m}$ = 1.0) under plane-wave illumination and for different values of the size parameter q. Linearly polarized light along the *x*-axis is incident from the bottom. Adapted with permission from [17]. Copyright 2017 Optical Society of America.

Several methods have been developed to overcome the diffraction limit and achieve maskless light-based nanopatterning, including near-field effects [9,10], multiphoton processes [11,12], laser interference [13,14], and use of stimuli-responsive materials [15,16]. More recently, photonic nanojets have shown promise as easy-to-use nanofabrication tools. Photonic nanojets consist of needle-like beams that originate at the backside of dielectric particles, such as glass spheres and cylinders [17], upon illumination. As shown in Figure 1a, placing the dielectric structure near or even in contact with a target substrate can result in localized material modification or ablation. Notably, the sub-diffraction nature of the jet allows the writing of nanometric features (Figure 1b). By scanning the nanojet relative to the target surface, arbitrary patterns can be generated, as shown in Figure 1c. 

While photonic nanojets have been demonstrated to be effective for subwavelength resolution and large-area nanopatterning, their use for bio-applications remains largely unexplored. A possible explanation is an existing gap between the scientific communities involved—biology and medicine, on the one hand, and physics and engineering, on the other. Here, with the idea of bridging this gap, we provide a broad overview of the underlying physics of photonic nanojets and their practical implementation for material processing. We emphasize aspects relevant to nanopatterning, including the width and length of a photonic nanojet, and the resulting intensity enhancement. Furthermore, we survey the strategies that have been so far implemented for generating arbitrary patterns (scanning) and increasing the throughput (parallelization) of photonic nanojet-mediated nanopatterning. Finally, we present successful uses of photonic nanojets for patterning substrates with tuned optical, mechanical, and electrical properties. They serve as an example of the potential of this technology for functional biomedical research.

## 2. Photonic Nanojet Formation and Properties

The formation of photonic nanojets occurs when illuminating dielectric particles immersed in a medium (typically air or water). It depends on the refractive index, shape, and size of the particles; the refractive index of the external medium; and the incident light wavelength. The conditions leading to nanojet formation can be obtained by directly solving Maxwell’s equations. Solutions are typically calculated by using approximated theories or numerical methods, such as finite-difference time-domain (FDTD) [18]. Alternatively, for spherical and cylindrical particles, Mie theory can be used. Readers interested in the details of the fundamental physics of photonic nanojets can refer to recent reviews on this subject [17,19]. Here, we restrict our analysis to the case of dielectric spheres. They are the most commonly used shapes, and the qualitative results obtained can be extrapolated to other geometries.

Nanojet formation in microspheres can be described using two main parameters: first, the relative refractive index *n*, defined as $n=n_{p}/n_{m}$, with $n_{p}$, and $n_{m}$ being the sphere and medium refractive indices, and second, the size parameter *q*, relating the particle size to the light wavelength (λ) and defined as $q=2\pi Rn_{m}/\lambda$, where R is the sphere radius. Depending on the values of n and q, different scenarios can be identified. Figure 1d summarizes the effect of the size parameter on the light fields generated by a glass microsphere in air (n = 1.5). If the sphere has a radius much smaller than the wavelength of light (q=0.1), the optical field resembles that of a dipole, with two clear lobes. As the sphere’s radius increases, the intensity distribution of the electric field becomes more pronounced in the forward direction (q=π and q=4π). When the sphere’s radius is much larger than λ, a narrow nanojet appears on the shadow side of the sphere (q=10π). Therefore, for a given refractive index ratio n, a nanojet is formed only above a specific size parameter. This result is valid for dielectric particles of other shapes, with the main difference being the particular threshold value of q that triggers the nanojet formation. For instance, for a glass ($n_{p}\;$= 1.5) microcylinder in air ($n_{m}$ = 1.0), a narrow nanojet at visible wavelengths forms for q~100.

The parameters n and q determine not only the condition for nanojet formation but also the light confinement achieved. In other words, the length and width of the nanojet and the resulting light enhancement depend on n and q. Thus, for a transparent microsphere ($n_{p}\;$= 1.33) surrounded by air ($n_{m}$ = 1.0) and under plane-wave illumination, the intensity enhancement, length, and width of the nanojets increase with the size parameter *q* [21]. For spheres of a given size, the nanojet properties can be tuned by altering the relative refractive index n. The simplest implementation of this strategy is varying the external refractive index, for instance, with liquids or transparent polymers. In general, an increase in the refractive index ratio shortens the nanojet and enhances its peak intensity, while slightly widening it—still remaining sub-diffracted for a wide range of index ratios. These trends, which in general apply to other particles, provide guidelines for selecting n and q based on the needs of a given application. As such, large *q* or high n will be favored for applications requiring high peak intensities, such as laser ablation, whereas large *q* and low n will lead to elongated nanojets suitable for patterning high-aspect-ratio nanostructures.

The above-discussed properties are valid for a dielectric particle surrounded by a homogeneous medium. For nanopatterning, however, this condition is altered by the proximity of the target substrate. In this case, effects such as light reflections or surface plasmon polaritons must be considered [22,23,24]. Under plane-wave illumination, the sphere-substrate system can act as a resonator that further contributes to the field enhancement [23]. In particular, the gap between the particle and the surface strongly modulates such enhancement; the smaller the separation gap, the stronger the field intensity. Therefore, for efficient material modification, the gap must be minimum-in several cases, the particles are placed in direct contact with the substrate.

## 3. Practical Considerations for Nanopatterning

One of the main advantages of nanopatterning systems based on photonic nanojets is simplicity. They require only three main elements: first, a light source and appropriate illumination conditions; second, suitable dielectric microparticles (at least one); and third, a mechanism for generating a geometric pattern into the sample surface, such as a method for scanning the nanojet relative to the surface, or an array of particles. In this section, these components will be discussed, emphasizing the aspects relevant for modifying materials and generating arbitrary patterns over large areas on a substrate.

### 3.1. Light Sources and Illumination Conditions 

Several light sources can be successfully used for photonic nanojet-mediated patterning, including UV lamps [25], light-emitting diodes (LEDs) [26], and lasers [27]. The choice between them depends, among other aspects, on cost, desired material modification, and optical properties of the substrate. UV lamps and LEDs are widely available, require minimal maintenance, and can be used for photolithography [28]. However, their broad emission spectra can result in chromatic aberrations that effectively widen the photonic nanojet. This problem can be obviated with dielectric microparticles that exhibit a low spectral dispersion. An alternative is making use of the inherent monochromaticity of lasers. Additional advantages of lasers are the possibility to select between continuous and pulsed sources. Continuous lasers have a relatively low cost—even comparable to LEDs—and enable ablation [27], as well as sintering [29] of highly absorbing materials. In contrast, pulsed lasers require an initial higher investment, but they can be used with a broader range of target materials. Indeed, sub-picosecond pulses enable the modification of transparent substrates [30,31]. Moreover, nonlinear absorption leads to light–matter interactions confined into the focal volume of the nanojet, further reducing the minimal feature size of the fabricated patterns [32,33]. Such a short interaction time can also reduce possible heating effects, thus increasing the pattern resolution [34].

Once the light source is chosen, other parameters, such as the angle, size, shape, and polarization of the incident light, can be used for controlling the properties of the nanojet. The angle of the incident light, in particular, controls the deflection of the nanojet, an option suitable for generating arbitrary nanopatterns as detailed in Section 3.3. The size of a collimated Gaussian beam—the most common beam shape at the output of commercial laser sources—relative to a microsphere dictates the intensity of the nanojet [35]. Additionally, a tightly focused Gaussian beam enables the control of the nanojet length, down to sub-diffraction values, by merely moving the focal position along the optical axis [36]. Note that the control of the beam waist or focal position of a Gaussian beam can be as simple as using a relay lens system or a focusing objective and a translation stage, respectively. The shape of the illumination beam also plays a crucial role in determining the properties of the light field at the output of the dielectric particle. For instance, a dielectric microsphere illuminated with a Bessel beam with a central lobe smaller than the sphere diameter does not result in a nanojet, but rather in a nanobeam exhibiting a bottle-like shape. Illumination with a doughnut and two half-circle beams produces a hollow nanobeam and a two-spot nanojet, respectively [37]. The polarization of the incident light can also lead to tailored light fields [38,39]. For example, annular nanobeams can arise from a dielectric microsphere when illuminated with an azimuthally polarized Gaussian beam [39]. 

### 3.2. Dielectric Microparticle Options 

As detailed in Section 2, the dielectric particles’ size and shape are fundamental to determining the properties of the resulting photonic nanojet. Therefore, careful attention must be paid to the selection of appropriate particles for a given application. Typically, dielectric microspheres are the preferred option. They provide high focusing power and are commercially available in different sizes, from nanometers to millimeters, and with materials ranging from silica to polystyrene. Unfortunately, photonic nanojets generated by highly focusing spheres exhibit a relatively short length—the jet interacts only with the substrate’s topmost portion. As a result, the height/depth of the feasible structures or the range of compatible substrate morphologies is limited.

**Figure 2:**
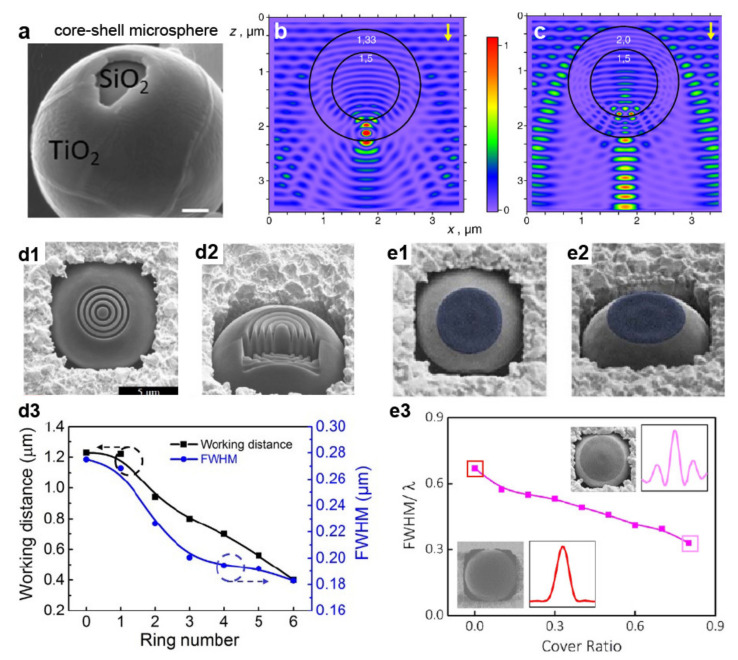
(**a**) Scanning electron micrograph (SEM) of a core-shell microsphere. Scale bar 100 nm. Reprinted with permission from [43] Copyright 2018 John Wiley & Sons, Inc. (**b**,**c**) Optical field distribution of core-shell microspheres with refractive index of the core 1.5 and that of the shell 1.33 (**b**) and 2.0 (**c**) under plane wave illumination. The field amplitudes are normalized to their maxima. Reprinted with permission from [21] Copyright 2010 Elsevier. (**d**,**e**) Surface-decorated microspheres. Top (**d1**) and side (**d2**) views of a microsphere decorated with four rings. (**d3**) Width and working distance versus ring number. Reprinted with permission from [47] Copyright 2015 Optical Society of America. Top (**e1**) and side (**e2**) views of a center-covered microsphere. In blue the cover. (**e3**) Nanojet width for various cover ratios. The insets show scanning micrographs and lateral intensity profile at the focal spot for cover-ratio of 0.0 and 0.777, respectively. Adapted with permission from [48] Copyright 2016 Springer Nature.

Further tunability of the nanojet properties can be achieved by using dielectric spheres with engineered refractive index profiles or surfaces. Core–shell microspheres are a good example of such an approach. In this case, the nanojet can be tuned by selecting the refractive index and radius of the core as well as the number, refractive index, and thickness of the shells [21,40]. As shown in Figure 2, two scenarios are possible for the case of a microsphere coated with a single shell. If the core’s refractive index is higher than that of the shell (Figure 2b), the curvature of the wavefront increases at the output of the microsphere. As a result, the length and width of the nanojet are smaller than they would be for the same microsphere without the shell. In contrast, if the core’s refractive index is smaller than that of the shell (Figure 2c), the wavefront’s curvature decreases, generating an elongated nanojet with a lower peak intensity. Numerical simulations indicate that ultra-narrow (width from λ/3.5 [41] to λ/7.3 [42,43]) as well as ultra-long (length from ~22 λ [44] to 100 λ [45,46]) nanojets can be generated with core-shell dielectric spheres and cylinders. 

Another method to tune the nanojet properties is decorating a microsphere’s surface to alter the transverse or longitudinal components of the electromagnetic field. Such a strategy can be implemented by etching concentric nanorings into the shadow surface of a silica microsphere (Figure 2(d1,d2)) [47]. In this case, the ring number tunes the working distance of the sphere and the nanojet width, reaching values as low as 0.485λ (Figure 2(d3)). Similar trends can be obtained by creating periodic corrugations into a dielectric microcylinder [49]. Alternatively, it is possible to block the central portion of the incident light, for example, by depositing a metallic circular mask (Figure 2(e1,e2)), with a radius r, directly onto the illuminated side of the sphere [48]. As shown in Figure 2(e3), in this design, the cover ratio r/R is key to engineer the nanojet, with the jet width progressively decreasing with this parameter. 

Despite the promise held by all the above-discussed approaches, reliable and precise control of a dielectric microsphere’s morphology and refractive index profile can be challenging. This has spurred the search for microparticles with different shapes for tailored nanojet formation, including solid immersion lens [50,51], ellipsoids [52,53], disks [54,55], toroids [56], cuboids [57,58], axicons [59,60], and even customized optical fiber tips [61,62]. Among them, microcuboids offer ease of fabrication and enable nanojets with tuned properties by simply selecting the cube size [63]. Axicons, on the other hand, can generate nanojets with a theoretical intensity enhancement (~40) close to that of optimized microspheres (~100) [64], but much longer (5.5 λ) [60]. Such elongated nanojets—they resemble Bessel beams—are attractive for patterning. They allow the preparation of high-aspect-ratio nanostructures and facilitate the processing complex and uneven topographies while avoiding focus axial translation [65]. Note that such nonconventional dielectric microparticles have not yet been used for material modification, offering new opportunities for researchers and scientists to expand the portfolio of applications suitable for nanojet-based nanopatterning.

### 3.3. Scanning Methods

**Figure 3:**
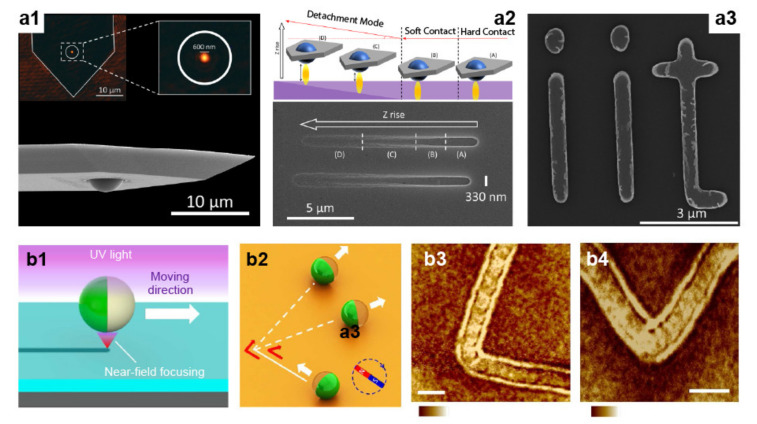
(**a1**) Scanning electron micrograph of an AFM cantilever with a microsphere. The inset is an optical image of the laser-illuminated sphere with the nanojet clearly visible. (**a2**) Schematic (top) and scanning electron micrograph (bottom) of out-of-plane scans. (**a3**) Scanning electron micrograph showing the letters IIT created with scanning probe photonic nanojet lithography. Adapted with permission from [68] Copyright 2017 American Chemical Society. (**b1**) Schematic showing a moving Janus microsphere motor near the photoresist surface under exposure to UV-light. (**b2**) Illustration of the magnetic guidance of sphere motion. (**b3,b4**) Patterns created by turning the magnetic field by 90° (**b3**) and 120° (**b4**). Scale bars 5 µm. Adapted with permission from [71] Copyright 2014 Springer Nature.

The strong light confinement achieved by a nanojet enables material modification at a precise location on a substrate. Still, generating a pattern requires point-by-point translation of the nanojet relative to a surface. The most common approach consists of physically moving the microparticle in the XY plane. Several methods have been developed to achieve such physical displacement, including mechanical [62,66,67,68], optical [20,69,70], and chemical forces [71]. Among the mechanical ones, mounting a microsphere into the cantilever of an atomic force microscope, or AFM, offers unsurpassed positioning control [68]. As shown in Figure 3(a1), in this setup, the piezoelectric stage of the AFM enables fine adjustment of the jet XY position (Figure 3(a2)), while the measurement of the cantilever deflection can be used as feedback to control the sphere-to-surface interspace. This mechanism allows the generation of regular and arbitrary patterns with subwavelength resolution (290 nm at λ = 405 nm), combining direct lithography and lift-off (Figure 3(a3)). A crucial step in this implementation is the attachment of the sphere to the cantilever, which has been achieved by using electrostatic forces and glues. 

Alternatively, optical traps can be used to displace the particle. McLeod et al. implemented this strategy by using a Bessel laser beam to trap dielectric microspheres in liquids [20]. Because of the perfect balance between the radiation force and the substrate’s electrostatic repulsion, the sphere accurately self-positions 50 nm apart from the surface without any active feedback control. This ability allows one to accommodate varying surface heights without refocusing the laser system and consequently patterning uneven or curved surfaces [72]. By using a nanosecond pulsed laser, coaxial with the Bessel beam, this method enables the ablation of arbitrary polymeric patterns with a minimum size of ~100 nm (<λ/3). The experimental setup can be simplified if the same pulsed laser is used for trapping and ablation [69]. For glass substrates, a post chemical etching of the laser-irradiated regions can be used to further reduce the minimal size to 70 nm (λ/11) [70]. Nanojet translation can also be achieved by means of chemical forces and Janus particles—particles half-coated with a metal, as shown in Figure 3b [71]. These particles propel autonomously in a specific direction if immersed in a chemical solution. Here, the propelling force originates from catalytic reactions at the metallic hemisphere, whereas the dielectric part forms a nanojet suitable for nanopatterning. The separation between the sphere and the surface is self-regulated by balancing electrostatic repulsions and van der Waals forces. For Janus microspheres decorated with a ferromagnetic layer, magnetic guidance can be used to further direct the trajectory of the nanojets and obtain precise nanopatterning (Figure 3(b2–b4)). 

**Figure 4:**
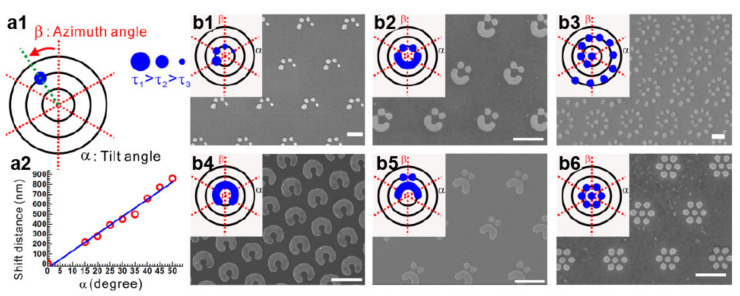
Nanojet lithography under oblique illumination (**a1**) Polar chart depicting three controllable variables (α, β, τ) for arbitrary nanopatterning. (**a2**) Linear relationship between the tilt angle (α) and shift distance of the focal position. (**b1**–**b6**) Examples of designed and generated periodic nanopatterns. Scale bars 1 μm. Adapted with permission from [73] Copyright 2017 American Chemical Society.

Patterning a substrate with photonic nanojets is also possible without physically translating the microparticle. In these cases, it suffices to deflect the nanojet. Such a strategy can be as simple as illuminating a dielectric particle at an angle [73,74]. For a microsphere, the incident angle controls the deflection’s extent almost linearly, as shown in Figure 4a [73]. Thus, nanometric shapes, such as spirals, circles, and dots, can be obtained by adjusting the tilt and azimuthal angles of the illumination along with the irradiation time [73]. Although such an approach provides flexibility in terms of the geometries that can be fabricated across the XY plane, deflecting the nanojets reduces the material modifications in the vertical direction, resulting in shallow 2D nanostructures. Deeper structures, such as vertical nanowires, can still be obtained by combining nanojet-mediated laser ablation with electrochemical etching [75]-the laser irradiation creates nucleation seeds for the chemical attack. In this case, the size, shape, and position of the nanostructures can be selected by modifying the illumination and sphere diameter, while the height only depends on the etching step. Alternatively, complex pseudo-3D morphologies can be directly defined with a single lithographic step by exploiting the lateral lobes of the optical field arising from the sphere [25,76]. However, the operational conditions—the intensity of the side lobes is effective for material modification when the particle size approaches λ—seriously constrain the nanojet properties, such as the intensity enhancement or length. Attempts to increase the pattern complexity by means of light interference, such as between light incidents into and scattered by the microsphere [77] or between adjacent nanojets [78], are promising, but control on the feasible geometries remains elusive. 

### 3.4. Nanopatterning with Parallel Photonic Nanojets 

The selection of different pattern geometries described in the previous section is based on the point-by-point irradiation of a surface and typically comes at the cost of a reduced throughput. In some instances, as in the case of bio-applications, it can be of interest to obtain higher throughputs even at the expense of partially sacrificing the ability to select the patterns. A straightforward method to increase processing throughput is exploiting multiple nanojets in parallel. Nanojet parallelization has been traditionally achieved by using an array of self-assembled microspheres [79,80]. In general, simple processes, such as spin coating, Langmuir–Blodgett, and template-assisted self-assembly [81], suffice to obtain a monolayer of hexagonally arranged spheres on top of a hydrophilic substrate [81]. The condition for nanojet formation is still valid when multiple arrayed microspheres are used, provided they have a large-size parameter. In this case, there is no mutual interaction between the light distributions arising from each sphere, and each of them generates its own nanojet. Therefore, processing throughput increases proportionally to the number of irradiated spheres. Notably, the field distribution formed by an array of spheres with a smaller size parameter, however, can still be predicted by using numerical methods, such as 3D FDTD or discrete dipole approximation [82,83,84]. Still, several drawbacks limit the efficacy of this approach. Only hexagonal arrangements are feasible, and the spatial resolution is limited to the sphere diameter. Because these methods apply only to hydrophobic surfaces, substrate selection is limited. Furthermore, the microspheres tend to detach from the substrate after irradiation, especially at high laser fluence as required for ablation, preventing multiple exposures. 

**Figure 5:**
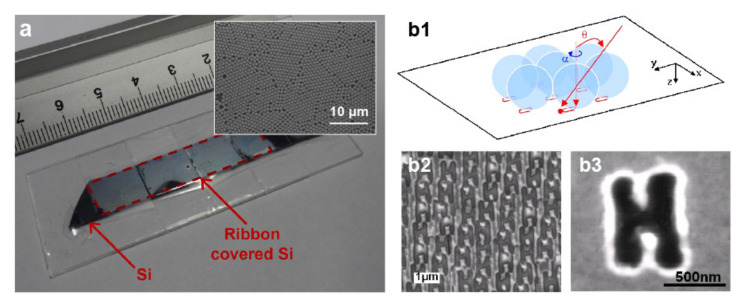
Parallelization of photonic nanojets. (**a**) Polypropylene substrate coated with acrylic adhesive for transporting microsphere array (referred to as the ‘Ribbon’) onto a Si surface. The inset shows a scanning electron micrograph of the array. Adapted with permission from [85] Copyright 2011 Elsevier. (**b1**) Schematic diagram of nanojet lithography under oblique illumination. (**b2**,**b3**) Scanning electron micrograph of H-shape arrays produced with tilted nanojets. Reprinted with permission from [88] Copyright 2009 Institute of Physics.

A method to address these issues is transferring the sphere array into a carrier substrate—typically a polymer coated with an adhesive. Such a strategy enables the placing of the microspheres directly onto hydrophobic surfaces, as shown in Figure 5a [85], and onto uneven morphologies [86]. Additionally, the adhesion forces between the carrier and the array can prevent the microspheres’ detachment after irradiation. Nonetheless, optical absorption in the UV or material heating at longer wavelengths caused by polymeric/adhesive carriers can limit the range of light sources suitable for nanopatterning. Alternatively, the sphere array can be prepared onto a glass substrate that, after being flipped upside down, is placed in direct contact with the substrate [87]. The resulting transportable array is transparent over a wide spectral range. It can also sustain multiple laser shots given the confinement of the microspheres between the substrate and the glass support. A problem still to be solved is the low versatility and resolution that sphere arrays provide. Figure 5b shows a possible solution consisting of irradiating the arrays under oblique illumination [88,89,90]. With this strategy, 6 × 10^6^ nanolines or arrays of alphabetic letters (Figure 5b) are simultaneously ablated in silicon over an area of 5 × 5 mm^2^ by multiple laser shots and angular scanning [90]. Because a relatively large area underneath the spheres can be patterned, the separation between adjacent structures can be reduced, thus also improving the spatial resolution. More recently, the combination of a digital micromirror device (DMD) with arrays of dielectric microparticles has proved effective in writing complex and parallelized patterns [29,91]. The gist of the approach is to individually illuminate selected microparticles of an array using the light positioning control offered by the DMD. Such combination shows promise for the direct laser ablation, sintering, or lithographic inscription of complex patterns over large areas (~cm^2^) while maintaining a subwavelength minimum feature size.

## 4. Nanopatterns for Biomedical Research

The interest in nanostructures for biomedical research is due to their unique optical, electrical, and mechanical properties that can be designed to meet the requirements of a target application. Nevertheless, the usage of nanostructured surfaces in bio-applications is limited by costly and small-area operations of current nanofabrication techniques. Via nanojet-mediated nanopatterning, tailored nanostructures can be fabricated at a high speed and with a simple and cheaper setup. Still, to consider nanojets as a convincing patterning tool for biomedical applications, it is necessary to ensure the functionality of the so-fabricated nanostructures. As detailed next, several results indicate that photonic nanojets can accomplish this task.

### 4.1. Enabling Results

Nanostructures for photonics. Photonic nanostructures offer the ability to control the interaction of light and molecules at the nanoscale. This is an essential feature for the development of highly sensitive analytical techniques, as required in important bio-applications ranging from clinical diagnosis to drug discovery. Within this framework, photonic nanojets can be a suitable tool for the fabrication of nanophotonic components based on plasmonic or photonic crystal structures.

**Figure 6:**
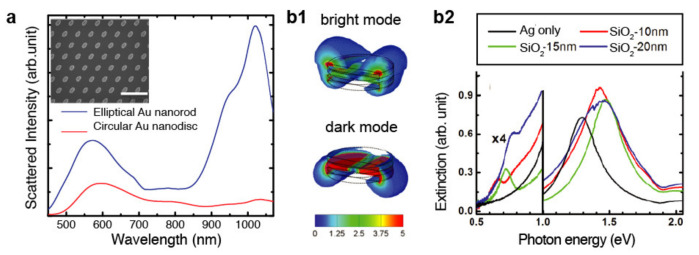
Nanostructures for photonics. (**a**) Measured dark-field scattering spectra of the circular and elliptical Au nanodisks fabricated with nanojet lithography under oblique illumination. The inset shows SEM images of the Au nanoellipses. Reprinted with permission from [97] Copyright 2012 The Japan Society of Applied Physics. (**b1**) Simulated energy field of the bright and dark mode of an Ag-SiO_2_-Ag dimer. (**b2**) Experimental extinction spectra of Ag-SiO_2_-Ag dimers with thickness 15 nm for Ag and 10, 15 and 20 nm for SiO_2_. The low energy side of the spectra highlights the shift of the dark plasmon mode when varying the SiO_2_ thickness. Adapted with permission from [94] Copyright 2012 American Chemical Society.

Nanojet lithography, followed by metal deposition and lift-off, allows the patterning of plasmonic nanostructures with a tailored spectral position and bandwidth of the resonance [92,93]. Several functional systems have been developed using this strategy, including arrays of metallic nanodots [92,93] and nanodisks [94]. They exhibit unique light–matter interactions, such as the ability to highly absorb light over large angles of the incidence, that is, to behave as perfect optical absorbers [92,95,96]. Notably, anisotropic nanostructures can also be prepared with nanojet lithography when using oblique illumination [97,98]. Such nanostructures exhibit longitudinal resonating modes that provide stronger light–matter interactions [99,100], which is useful for biosensing and chemical analysis [101,102]. For instance, tilted nanojets generate elliptical spots that, once recorded into the photoresist, translate into metallic nanoellipses by lift-off. As shown in Figure 6a, the so-fabricated gold nanoellipses show two spectral peaks associated with the transverse mode along the disk thickness and the longitudinal mode along the major axis of the ellipse, respectively. Another example of anisotropic structures fabricated with nanojets are plasmonic dimers, namely, couples of metal nanostructures in close proximity (gap < 100 nm) [103]. By placing two Ag nanodisks with a SiO_2_ layer in between, dimers exhibiting two resonances corresponding to the characteristic dark and bright modes of such structures have been reported [94] (Figure 6(b1)). Importantly, nanojet lithography enables the tuning of such resonances by controlling the SiO_2_ thickness (Figure 6(b2)). 

Photonic crystals-materials with a periodic refractive index modulated at the micro- [104] or nanoscale [105]-can also be fabricated using photonic nanojets. Specifically, polymeric 2D photonic crystals with a selectable lattice and, hence, optical properties can be prepared by combining multiple UV exposures with oblique and off-axis illuminations [106]. In this case, patterns typically consist of periodic nanoholes surrounded by a photoresist. The key parameter of a photonic crystal is the refractive index contrast of the composing materials; the higher the index contrast, the wider the transmission bandgap [107,108]. However, the use of conventional photoresists as a structural material, with a refractive index in the range of 1.4–1.5, limits the refractive index contrast of the crystal. Such an effect is especially noted for operations with liquids, as required for (bio)chemical sensing—the nanoholes are filled with liquids with a refractive index above 1.3. To address this issue, photoresists with a higher refractive index have been used, such as titanium dioxide based sol−gel resists with a refractive index between 1.65 and 2.25 [109]. 

Photonic nanojets can also be used for the nanofabrication or surface decoration of LEDs. A possible method is patterning the surface of a conventional LED with nanostructures intended to improve the light extraction and quantum efficiency of the original devices [110]. By using nanojet lithography and multiple exposures at various incidence angles, a gallium nitride LED is decorated with selectable 2D photonic crystal structures. Compared with the original LED, the light output power of the modified device was 44.85% higher. Another method is using nanojets for the direct fabrication of nanostructured LEDs [111,112]. Chou et al. reported on the emission of polarized light with III-nitride elliptical nanorod LEDs prepared by photonic nanojet lithography and post chemical etching [113]. The measured electroluminescence was found to be the highest for polarization parallel to the major axis of the ellipse.

*Nanostructures for electronics.* Electrical processes play a fundamental role in living systems. They affect neuronal communications and the binding affinity of biomolecules. As such, there is a growing interest in micro- and nanoelectronic devices capable of detecting a large variety of bioanalytes-nucleotides, amino acids, and even cells-for applications as critical as rapid diagnostics, genetic screening, and personalized medicine.

**Figure 7:**
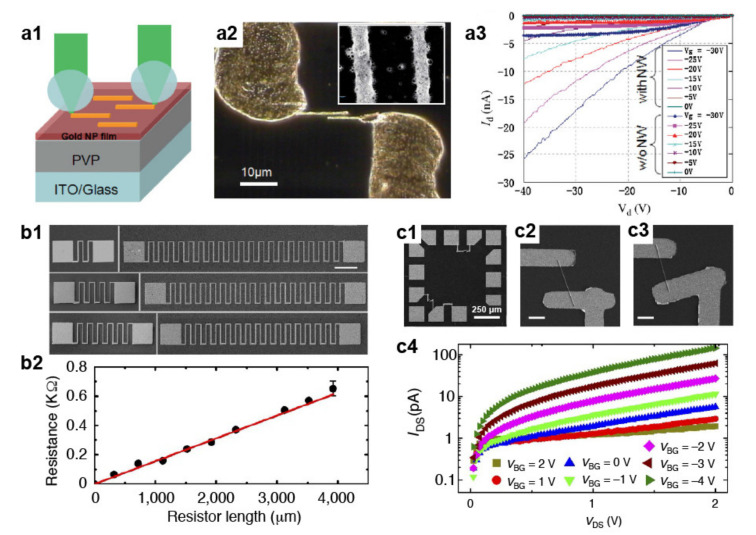
Nanostructures for electronics. (**a1**) Illustration of the fabrication of nanotransistors by means of nanojet-mediated laser sintering. (**a2**) Optical dark-field and SEM images of sintered nanowires (NWs). (**a3**) Characteristic I–V curves of the OFET with and without NWs. Adapted with permission from [29], Copyright 2010 John Wiley & Sons, Inc. (**b1**) Serpentine resistors patterned by exploiting an array of individually addressable hollow pyramids. Scale bar 100 µm. (**b2**) Plot of the resistance versus resistor length (black dots) with linear fitting (red line). (**c1**–**b3**) SEM image of electrode pads with the nanowires. The scale bar 5 µm. (**c4**). I–V curves for different gate voltages, highlighting the characteristic transistor behavior. Adapted with permission from [91], Copyright 2013 Springer Nature.

Recent results indicate that photonic nanojets can be used for the fabrication of several nanoelectronic components. Pan et al. reported on the high-throughput nanofabrication of organic field-effect transistors, or OFETs, using multiple photonic nanojets formed by individually addressable microspheres [29]. The intensity of each nanojet was used to sinter pairs of nanowires from a thin film of colloidal gold nanoparticles deposited onto an ITO substrate. Each pair, with a width of 250 nm and separated 800 nm apart, served as the drain and source electrodes of an OFET, as shown in Figure 7a. By using hollow micropyramids instead of microspheres, nanoresistors with ohmic behavior (Figure 7b), inductors, interdigitated capacitors, and nanowires-based FETs (Figure 7c) were prepared as well [91]. 

Nanofabrication of p-n and Schottky junctions is another possible application of photonic nanojets to nanoelectronics [114,115]. For instance, nanojet lithography and electrodeposition techniques can be used to prepare arrays (~cm^2^) of cadmium telluride (CdTe) nanorods on top of ITO [114]. More recently, molybdenum disulfide/gold or MoS_2_/Au Schottky junctions have been fabricated by nanojet lithography and lift-off [115]. Each junction consisted of close-packed Au disks on top of a MoS_2_ layer. By adjusting the exposure, the disk size was controlled. The integration of the MoS_2_/Au junctions into a MoS_2_ transistor led to eightfold photoresponsivity enhancement due to the extra hole traps that the nanojunctions provided.

*Mechanical nanostructures*. Nanostructured substrates with enhanced mechanical properties are central components of systems, such as wearable sensors and scaffolds mimicking the mechanical stimuli of in vivo cells. In these instances, nanojet-mediated lithography can be a suitable fabrication tool [116]. For instance, a monolayer of close-packed polystyrene spheres (diameter 500 nm) was used for nanopatterning a photoresist template, which was then conformally coated with either zinc oxide (ZnO) or alumina (Al_2_O_3_) by means of atomic layer deposition. The subsequent removal of the resist led to shell nanostructures consisting of vertically aligned nanotubes. The thickness of the oxide layer controls the nanostructures’ density. Thus, the mechanical properties of the system, including ductility and fragility, become tunable. In particular, nanoindentation tests under cyclic incremental loads revealed that only the thinnest shells exhibited a ductile behavior characterized by a record elastic modulus of 1.19 GPa, a specific energy dissipation of 325.5 kJ/kg, and a hardness of 7.8 MPa 

### 4.2. Nanostructures for Bio-Applications

Among the many bio-applications that could benefit from photonic nanojet-mediated nanopatterning, biosensing is the one that has been overwhelmingly exploited so far. The extensive results on the nanofabrication of plasmonic structures stimulated the usage of photonic nanojets for patterning substrates suitable for surface-enhanced spectroscopy, specifically, surface-enhanced Raman scattering (SERS) and surface-enhanced infrared absorption (SEIRA). For these techniques, the large parameter space regarding the geometry and size that nanojets provide is key to achieving excellent detection capabilities. Indeed, the spectral position and bandwidth of the plasmon resonances depend on the size and shape of the nanostructures [93,117]. Therefore, strong field enhancements and high sensitivity can be reached when the resonance matches a molecule’s vibrational mode. Furthermore, by promoting the capture of a large number of target molecules, anisotropic or high-aspect-ratio nanostructures enable the detection at high signal–noise ratios and, hence, at low concentration limits. 

SERS substrates with high sensitivity can be prepared by parallelized nanojet-mediated laser ablation of silicon followed by chemical etching and Ag deposition [118]. In particular, the ablation of a Si substrate can result in periodic micropits that after alkali etching mutate into nanovolcanoes whose spacing, widths, and heights can be tuned with the sphere diameter, laser power, and etching time. After Ag deposition, novel plasmonic flowerlike nanostructures form on top of the volcanoes. The resulting SERS substrates are functional for molecular analysis. In particular, they enabled the detection of 1 nM of rhodamine 6G (R6G) and 10 ppm of fenthion, a moderately toxic compound typically used as pesticide. Interestingly, the substrate exhibited a field enhancement as high as 8.2 × 10^7^ for R6G, which was ascribed to the plasmon mode of the ablated Si structures. This is consistent with the broadband enhancement (>2) of the Si Raman mode previously observed in nanoholes prepared by photonic nanojet-mediated laser ablation [119].

**Figure 8:**
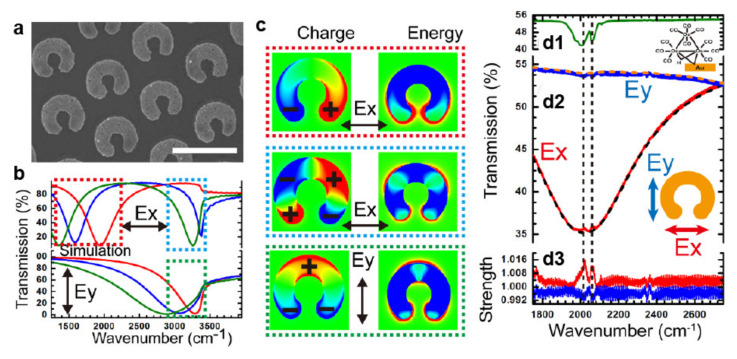
SEIRA spectroscopy. (**a**) SEM image of C-ring Au nanodisks prepared by multi-exposure angled nanojet lithography. (**b**) Theoretical polarized transmission spectra of C-rings for outer diameters of 560 nm (red lines), 600 nm (blue lines), and 680 nm (green lines). (**c**) Charge and field energy distributions of the resonance modes for various polarization states. (**d**) Standard transmission spectrum of osmium carbonyl clusters (molecular structure in the inset) (**d1**). (**d2**) Measured transmission spectra and (**d3**) vibrational strength of the C-ring array with osmium carbonyl clusters excited with Ex and Ey polarizations. Black and gold curves are baselines. Adapted with permission from [73], Copyright 2017 American Chemical Society.

The strong surface enhancement of SERS substrates often leads to fluorescence emission, which hampers the detection of the Raman signature of the target molecules. SEIRA substrates mitigate the problem because they exhibit lower enhancement factors (<10^3^) but are still sufficiently high for (bio)chemical analysis [120]. Chien et al. reported on SEIRA substrates prepared by combining nanojet lithography, under oblique illumination, with angled metal deposition and lift-off [73]. As shown in Figure 8, the array consisted of periodic gold C-rings and exhibited polarization-dependent plasmonic resonances tunable with the ring’s outer diameter. In particular, the nanostructure sustained dipole and quadrupole modes depending on the polarization of the exciting light. The SEIRA substrates were tested for detecting the signature of osmium carbonyl clusters, a compound used in immunoassays and pharmaceutics, in the IR between 1500 and 3000 cm^−1^. The transmission spectrum of osmium carbonyl clusters (Figure 8(d1)) revealed double peaks near 2000 cm^−1^ corresponding to various carbonyl-stretching vibrations. The strength of the SEIRA signals, for X- and Y-polarized exciting light, significantly increased when the vibrational modes matched the plasmonic resonance of the rings (Figure 8d2,d3). 

### 4.3. Prospective Bio-Applications

The high reliability, resolution, competitive cost, and throughput of nanojet-mediated nanopatterning can open novel opportunities in biomedical applications and consolidate established ones. An intriguing possibility is the nanofabrication of optical label-free biosensors. Among the current approaches, photonic crystals [101,121] and plasmonic resonators [122,123] have proved excellent sensitivity and detection limit when used for molecular recognition. Specifically, they provide unsurpassed detection of the refractive index variations produced by molecules binding into the sensor surface. Photonic nanojet lithography and ablation offer a cost-effective alternative to existing nanofabrication methods for producing optical nanosensors at the wafer scale. Arguably, this is the area where photonic nanojets are posed to make the most significant impact.

Besides optical methods, photonic nanojet may enable the nanofabrication of biosensors based on field-effect transistors (BioFETs), namely, FETs gated by changes in the surface potential due to the binding of molecules onto the gate electrode [124,125]. Among possible implementations, BioFET-exploiting nanostructured gates (nanowires, nanotubes, graphene) have attracted significant attention for bio-applications requiring ultrasensitivity and rapid response [126,127,128]. However, the high device-to-device variability and the absence of mass production nanofabrication technology are currently limiting the commercialization of nanostructured BioFETs. By using nanojet lithography, which has already been proven capable of producing nanotransistors, these issues could be addressed. This scenario could also be of interest for other biomedical applications, such as portable diagnostic systems (lab-on-chip) or wearable sensors (ECG, glucose sensors), for which miniaturized electronics and power sources are essential components. 

Another potential biomedical application is drug delivery. Several works have shown that micro- and nanostructures (pillars, needles) can penetrate the skin, cells, or other tissues, enabling molecular delivery and biological therapy [129,130,131]. However, for some applications, such as cell transfections, even small variations in the geometries of the nanostructures can result in a significant change in the cell response. This problem is particularly serious when the nanostructures occupy large areas because heterogeneous cell responses lead to scarce delivery efficiency and results difficult to interpret. Still, in the field of cellular biology, nanostructured surfaces regulate cell migration, proliferation, and differentiation [132,133,134]. For these applications, both the morphology (size and geometry) and physical properties (wettability, stiffness) of the substrates have an impact on cellular behavior. The wide range of compatible materials (polymers, glass, silicon) and morphological options (holes, pillars) that photonic nanojets enable offer remarkable chances to precisely engineer cell responses. These opportunities can be extended to other living organisms, such as bacteria [135], algae [136], and fungi [137], whose proliferation is of interest for several bio-applications, including the study and treatment of chronic bacterial infections. 

Finally, a field of great interest for biomedical research is micro- and nanofluidics. Fluidic systems at such scale enable operations as important as separation, concentration, manipulation, and detection of (bio)molecules at higher sensitivity and throughput and lower costs than conventional lab-scale methods [138,139]. A typical example is DNA separation—a crucial step in cellular and molecular biology—which can be efficiently performed by exploiting the unique dynamics of fluids flowing into a nanochannel. Unfortunately, these technologies face fundamental challenges given the limitations of traditional micro- and nanofabrication methods regarding material availability and minimum feature size. Photonic nanojets can offer new avenues of opportunity to push fluidics to the nanoscale and advance toward the next generation of biomedical devices.

## 5. Conclusions

The sub-diffractive nature of photonic nanojets enables material modification at a sub-micrometric scale. The ability to precisely nanostructure a surface at a high throughput and with a simple instrument opens the door to the consolidation of nanotechnology not only into traditional fields such as nanophotonics or nanoelectronics, but also into less explored ones, such as biomedical research. Nanojets have been successfully applied to lithography, polymerization, laser ablation, and sintering, thus providing a large degree of flexibility regarding materials and substrate options. Selection of the operational parameters, including illumination and dielectric microparticles, allows engineering jets with tailored length, width, and intensity enhancement to meet the requirements of a specific material or application. Recent progress in nanojet parallelization, together with optimized illumination schemes, enables the coverage of large areas of a surface with complex nanostructures. Still, the generation of arbitrary patterns remains an issue to be addressed. Strategies used so far surely provide flexibility in terms of pattern selection but often at the cost of an increased complexity of the experimental setup. Therefore, future works should be devoted to expanding the range of patterning geometries that nanojets offer without sacrificing their rare implementation simplicity. 

We believe that the potential applications of nanojet-mediated nanopatterning are not limited to the examples surveyed in this work. For instance, light-activated synthesis of emerging materials (perovskite, conductive polymers, 2D materials [140,141,142]) may help achieve significant advances in several fields as important as energy harvesting, sustainable electronics, and wearable sensors. Furthermore, the possibility of exploiting photonic nanojets directly for optical sensing or surgery may further expand and complete their portfolio of biomedical applications [143,144]. As photonic nanobeams with novel features are engineered—photonic nanohook is a quite recent example [145]-and innovative illumination methods [146,147] are implemented to enhance the light–matter interaction, interesting applications for research and industry will continue to emerge.
